# Engineering Clostridial Aldehyde/Alcohol Dehydrogenase for Selective Butanol Production

**DOI:** 10.1128/mBio.02683-18

**Published:** 2019-01-22

**Authors:** Changhee Cho, Seungpyo Hong, Hyeon Gi Moon, Yu-Sin Jang, Dongsup Kim, Sang Yup Lee

**Affiliations:** a Metabolic Engineering National Research Laboratory, Systems Metabolic Engineering and Systems Healthcare (SMESH) Cross-Generation Collaborative Laboratory, Department of Chemical and Biomolecular Engineering (BK21 Plus Program), Center for Systems and Synthetic Biotechnology, Institute for the BioCentury, Korea Advanced Institute of Science and Technology (KAIST), Daejeon, Republic of Korea; b Research Division of Food Functionality, Korea Food Research Institute, Wanju-gun, Jeollabuk-do, Republic of Korea; c Department of Bio and Brain Engineering, KAIST, Daejeon, Republic of Korea; d BioProcess Engineering Research Center, KAIST, Daejeon, Republic of Korea; e BioInformatics Research Center, KAIST, Daejeon, Republic of Korea; University of Massachusetts Amherst; University of Illinois at Urbana-Champaign; Chinese Academy of Sciences

**Keywords:** *Clostridium acetobutylicum*, aldehyde/alcohol dehydrogenase, butanol selectivity, metabolic engineering, protein engineering

## Abstract

Butanol production by Clostridium acetobutylicum is accompanied by coproduction of acetone and ethanol, which reduces the yield of butanol and increases the production cost. Here, we report development of several clostridial aldehyde/alcohol dehydrogenase (AAD) variants showing increased butanol selectivity by a series of design and analysis procedures, including random mutagenesis, substrate specificity feature analysis, and structure-based butanol selectivity design. The butanol/ethanol ratios (B/E ratios) were dramatically increased to 17.47 and 15.91 g butanol/g ethanol for AAD_F716L_ and AAD_N655H_, respectively, which are 5.8-fold and 5.3-fold higher than the ratios obtained with the wild-type AAD. The much-increased B/E ratio obtained was due to the dramatic reduction in ethanol production (0.59 ± 0.01 g/liter) that resulted from engineering the substrate binding chamber and the active site of AAD. This protein design strategy can be applied generally for engineering enzymes to alter substrate selectivity.

## Abstract (precis)

Renewable biofuel represents one of the answers to solving the energy crisis and climate change problems. Butanol produced naturally by clostridia has superior liquid fuel characteristics and thus has the potential to replace gasoline. Due to the lack of efficient genetic manipulation tools, however, clostridial strain improvement has been slower than improvement of other microorganisms. Furthermore, fermentation coproducing various by-products requires costly downstream processing for butanol purification. Here, we report the results of enzyme engineering of aldehyde/alcohol dehydrogenase (AAD) to increase butanol selectivity. A metabolically engineered Clostridium acetobutylicum strain expressing the engineered aldehyde/alcohol dehydrogenase gene was capable of producing butanol at a high level of selectivity.

## INTRODUCTION

Biology-based production of chemicals and fuels from renewable biomass has been attracting much attention to address climate change and other environmental problems (1–3). In the biofuel sector, ethanol has been most widely used because of its long history of availability, but butanol has recently been receiving renewed interest because its air-to-fuel ratio and energy density are similar to those of gasoline and because it is less hygroscopic (4–6). Also, butanol is an important industrial chemical used to make butyl acrylate, methacrylate esters, butyl glycol ether, and butyl acetate (6). Butanol is naturally produced by several clostridial strains through the well-known process of acetone-butanol-ethanol (ABE) fermentation. Among butanol-producing strains, Clostridium acetobutylicum has been extensively studied as a model organism for ABE fermentation (7–10). The typical fermentation by C. acetobutylicum yields acetone, butanol, and ethanol at the weight ratio of 3:6:1 (7). Coproduction of acetone and ethanol results in reduced butanol yield and increased recovery cost, causing increased butanol production cost (7).

There have been several studies on improving butanol selectivity rather than reducing acetone and ethanol production. C. acetobutylicum strain M5, which does not possess the pSOL1 megaplasmid carrying the essential genes for butanol and acetone formation, has been used as a platform strain to produce butanol efficiently (11, 12). Using the C. acetobutylicum M5 strain, butanol production could be restored by expressing the *adhE1* gene (11) or the *adhE1* and *ctfAB* genes (12). Overexpression of the *adhE1* gene under the control of the *ptb* promoter in C. acetobutylicum strain M5 showed a butanol/ethanol (B/E) ratio of 4.34 g/g without acetone production (11), while overexpression of the *adhE1* and *ctfAB* genes showed a B/E ratio of 10.0 g/g with a small amount of acetone (12). In a recent study, six aldehyde/alcohol dehydrogenases (*adhE1*, *adhE2*, *bdhA*, *bdhB*, *SMB_P058*, and *yqhD*) of C. acetobutylicum DSM 1731 were evaluated with respect to butanol and ethanol production through inactivation of the corresponding genes (13). Among the six aldehyde/alcohol dehydrogenases, inactivation of the *adhE2* gene resulted in a B/E ratio (8.15 g/g) that was higher than that seen with the wild-type strain (6.65 g/g). Furthermore, both the *SMB_P058* mutant and *yqhD* mutant produced less ethanol without loss of butanol formation, which led to higher B/E ratios of 10.12 g/g and 10.17 g/g, respectively (13).

However, the use of metabolic engineering approaches alone has shown limitations in further increasing the butanol selectivity. The key enzyme in butanol production is aldehyde/alcohol dehydrogenase (AAD), which is encoded by the *adhE1* gene residing on the pSOL1 megaplasmid (13). This enzyme comprises an aldehyde dehydrogenase (ALDH) and an alcohol dehydrogenase (ADH), which converts butyryl-coenzyme A (butyryl-CoA) to butyraldehyde and butyraldehyde to butanol, respectively (14). The metabolic network of C. acetobutylicum has been engineered to increase fuel production by the use of different strategies such as reduction in acetone production and conversion of acetone into isopropanol (11, 12, 15–17). However, it has been rather difficult to increase the B/E ratio by such metabolic engineering approaches, mainly because AAD also converts acetyl-CoA to ethanol. Thus, it is essential to engineer AAD to increase butanol selectivity and reduce ethanol production. It was previously reported that introducing three mutations in Zymomonas mobilis ADH2 increased the enzyme activity with respect to butanol by increasing hydrophobicity at the substrate binding site (18). Inspired by this work, we examined whether it is possible to engineer C. acetobutylicum AAD for achieving high butanol selectivity.

## RESULTS

### Developing AAD variants based on Z. mobilis ADH2 characteristics.

**FIG 1:**
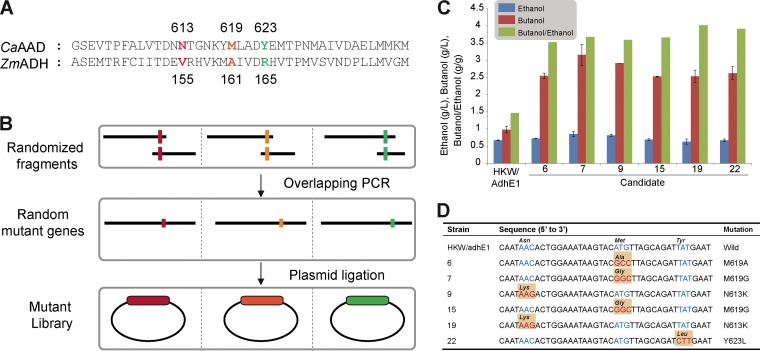
Screening of butanol selectivity. (A) Alignment of C. acetobutylicum AAD and Zymomonas mobilis ADH2, which harbors butanol selectivity mutants on residues V155, A161, and R165. (B) Construction of mutant library for butanol variants. (C) Variants with increased butanol selectivity. (D) Sequence characterization of butanol selectivity mutants.

In the first attempt, we randomized three residues, N613, M619, and Y623, which correspond to the three mutations in Z. mobilis ADH2 responsible for higher butanol activity. Using a pTHL1-Cm expression plasmid allowing gene expression under the control of the strong constitutive thiolase promoter, plasmids pTHL1-MU613, pTHL1-MU619, and pTHL1-MU623 (harboring random mutations at the N613, M619, and Y623 positions in the *adhE1* gene, respectively) were transformed into the C. acetobutylicum HKW strain, in which the native *adhE1* gene was inactivated (19) (Fig. 1A and B; see also Fig. A in Text S1 in the supplemental material). When these transformants were cultured in the 24-well microtiter plates containing 2 ml of clostridial growth medium (CGM), 6 candidates among about 500 colonies showed B/E ratios ranging from 0.8 to 1.8 g/g, which were values higher than that obtained with the control strain (B/E ratio of 0.8 g/g). When the HKW strains harboring plasmids for these six mutant *adhE1* genes were cultured in capped serum bottles for 48 h, the B/E ratios were 2.4-fold to 2.7-fold higher than the ratios obtained with the control strain (Fig. 1C). Sanger sequencing of the six AAD variants revealed the following mutations: M619A, M619G, N613K, M619G, N613K, and Y623L (Fig. 1D). Since two variants showed the same mutations, four unique mutations, M619A, M619G, N613K, and Y623L, were found to be beneficial mutations contributing to higher butanol selectivity.

10.1128/mBio.02683-18.1 TEXT S1 Supplemental figures and tables. Download Text S1, DOC file, 1.4 MB. Copyright © 2019 Cho et al. 2019 Cho et al. This content is distributed under the terms of the Creative Commons Attribution 4.0 International license.

### Increased butanol selectivity of mutant AADs in C. acetobutylicum M5.

In order to examine the effects of these mutant AADs on ethanol and butanol formation, mutant plasmids pTHL1-N613K, pTHL1-M619A, pTHL1-M619G, and pTHL1-Y623L were transformed into the C. acetobutylicum M5 strain, which does not harbor the megaplasmid pSOL1 and thus does not produce acetone and butanol. A typical batch fermentation of M5 strain resulted in production of 0.60 ± 0.02 g/liter of ethanol, together with 3.37 ± 0.22 and 24.10 ± 0.02 g/liter of acetic and butyric acids, respectively. Butanol production was slightly increased to 10.71 to 11.66 g/liter in recombinant M5 strains expressing four mutant AADs compared with the level (10.01 ± 0.12 g/liter) obtained with the M5 strain harboring the wild-type AAD in batch fermentation (Table 1; see also Fig. B in Text S1). On the other hand, ethanol production was considerably decreased to 1.07 to 2.63 g/liter in the strains expressing four mutant AADs, while the strain harboring the wild-type AAD produced 3.31 ± 0.01 g/liter of ethanol (Table 1). The B/E ratios obtained with the M5 strains expressing four mutant AADs were 4.17 to 9.72 g/g, which were higher than that (3.02 g/g) obtained with the M5 strain expressing the wild-type AAD (Table 1). These results suggest that single amino acid substitutions on AAD indeed affected the substrate (acetaldehyde or butyraldehyde) binding site and consequently increased butanol selectivity.

**TABLE 1:** Comparison of fermentation results obtained with Clostridium acetobutylicum strain M5 and engineered strains^*a*^

				Titer (g/liter)							
Strain	pH	Temp (°C)	OD_600_	Butanol	Acetone	Ethanol	Butyrate	Acetate	Butanol yield^*b*^ (g/g)	Butanol selectivity^*c*^ (g/g)	B/E ratio^*d*^ (g/g)
M5	5.5	37	13.7	0.14 ± 0.01	0	0.60 ± 0.02	24.10 ± 0.02	3.37 ± 0.22			
M5 (pTHL1-AdhE1)	5.5	37	12.9	10.01 ± 0.12	0	3.31 ± 0.01	0.95 ± 0.00	8.58 ± 0.21	0.158	0.75	3.02 ± 0.03
M5 (pTHL1-N613K)	5.5	37	11.7	11.66 ± 0.01	0	1.68 ± 0.00	3.45 ± 0.29	9.28 ± 0.23	0.178	0.87	6.92 ± 0.01
M5 (pTHL1-M619A)	5.5	37	15.3	11.40 ± 0.01	0	2.32 ± 0.01	1.27 ± 0.59	9.25 ± 0.94	0.161	0.83	4.83 ± 0.04
M5 (pTHL1-M619G)	5.5	37	15.6	10.71 ± 0.00	0	1.07 ± 0.00	1.83 ± 0.01	6.48 ± 0.01	0.190	0.91	9.72 ± 0.00
M5 (pTHL1-Y623L)	5.5	37	13.8	11.01 ± 0.01	0	2.63 ± 0.01	0.76 ± 0.09	9.49 ± 0.52	0.161	0.81	4.17 ± 0.02

a Batch fermentations were conducted at least in duplicate for reproducibility checks.

b Butanol yield, butanol produced divided by glucose consumed (gram/gram).

c Butanol selectivity, ratio (gram/gram) of butanol to total solvents.

d B/E, ratio (gram/gram) of butanol to ethanol.

### Structural models of butanol selectivity ADHs.

**FIG 2:**
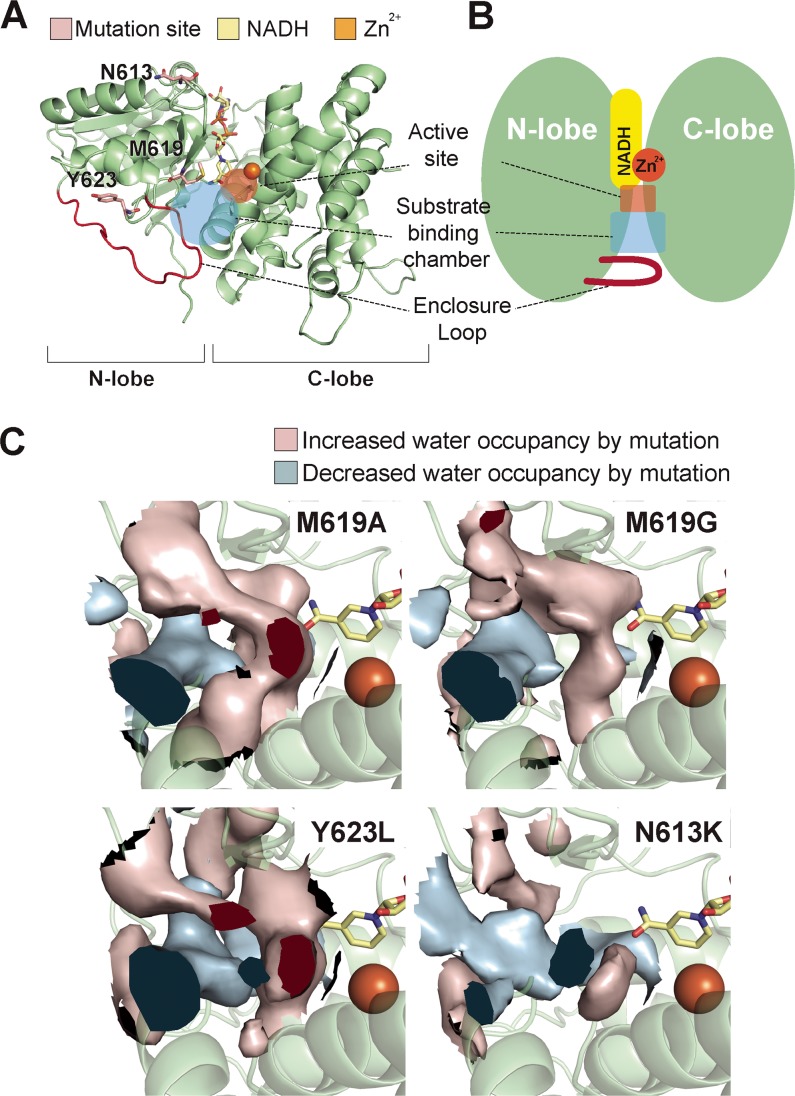
Structural model of ADH and three mutation residues. (A) ADH of C. acetobutylicum is composed of an N-terminal domain (NTD) and a C-terminal domain (CTD) forming two clefts between them. Zn^2+^ is placed at the center of the enzyme; NADH occupies one cleft, and the other cleft forms a substrate binding chamber. All three mutation residues are located on the NTD. M619 is placed on the substrate binding chamber. N613 is placed on the tip of loop that is associated with beta strands, which form a substrate binding chamber. Y623 is located at the hydrophobic core that stabilizes α helix and loop structures. The mutation residues are represented as pink sticks. (B) Schematic representation of ADH structure. (C) Increased water occupancy in substrate binding chamber for butanol selectivity mutations. The average levels of water occupancy around ADH were calculated from molecular dynamic simulation trajectories. The difference in the levels of water occupancy between butanol selectivity mutants and the original structure was computed. The change in the level of water density that resulted from the mutations indicates that the water occupancy levels in the substrate binding chambers had been increased. Increases in the levels of water occupancy in wide regions of the substrate binding chambers in AAD_M619A_, AAD_M619G_, and AAD_Y623L_ can be clearly seen. The increase of the level of water density was small for the N613K mutations and was located near the active site.

In order to further understand the mutational effects of butanol selectivity mutations, structural models of the ADH portions of the wild-type AAD and the four mutant AADs were constructed and examined for structural features associated with the increased butanol selectivity. Homology modeling was performed based on the crystal structure of the Geobacillus thermoglucosidasius ADH, which showed 48% amino acid sequence identity with the C. acetobutylicum ADH (see Table S1 in the supplemental material). This ADH domain is composed of N- and C-terminal lobes harboring NADH and Zn^2+^ and substrate binding sites between their interfaces (Fig. 2A and B). The structures of C. acetobutylicum and G. thermoglucosidasius ADHs are distinctively different from those of the other well-known alcohol dehydrogenases in that the substrate binding site is enclosed by a loop extended from the N-terminal lobe (Fig. 2A; see also Fig. S1 in the supplemental material). The four mutations leading to higher butanol selectivity were located on the N-terminal lobe (Fig. 2A). The M619 residue constitutes the lining of the substrate binding chamber, implying direct involvement in substrate binding, but residues N613 and Y623 are located outside the substrate binding chamber. In previous works, substrate selective mutations were mostly located near the active sites and in the substrate binding site, but some were distant from the active site (18, 20) (see Fig. S2), implying that the mutational effect can be either direct or indirect. We then performed molecular dynamics simulations with the wild-type and mutant AAD structures to investigate the mutation-induced structural changes shared by the butanol selectivity mutants, especially at the substrate binding chamber.

10.1128/mBio.02683-18.2 FIG S1 Substrate passage of ADHs. (A and B) The structure of C. acetobutylicum ADH was modeled using the structure of G. thermoglucosidasius ADH. Unlike other ADHs, there is an extended loop in both ADHs. (C and D) The loop is placed at the passage to the active sites, which was found by the use of CAVER 3.0 (E. Chovancova, A. Pavelka, P. Benes, O. Strnad, J. Brezovsky, B. Kozlikova, A. Gora, V. Sustr, M. Klvana, P. Medek, L. Biedermannova, J. Sochor, and J. Damborsky, 2012, PLoS Comput Biol 8:e1002708). In addition, there is another shorter passage to the active sites in the C. acetobutylicum ADH. (E and F) The obstruction of a longer passage was not found in Z. mobilis ADH2 (PDB ID = 3OX4). (G and H) A shorter passage was found in another enzyme with NADH, namely, carbonyl reductase (PDB ID = 4C4O). Due to large structural differences, the structures of NAD were superimposed on that of Z. mobilis ADH2. Download FIG S1, TIF file, 48.9 MB. Copyright © 2019 Cho et al. 2019 Cho et al. This content is distributed under the terms of the Creative Commons Attribution 4.0 International license.

10.1128/mBio.02683-18.3 FIG S2 Substrate specificity mutations. (A) Substrate specificity of Z. mobilis ADH2. (B) Horse liver ADH. (C) Yeast ADH. (D) Mutations reducing enzymatic activity for 2-butanol and 2-pentanol in Sulfolobus solfataricus ADH. (E) Stereo-specificity mutations in Thermoanaerobacter brockii ADH. (F) Stereo-specificity mutations in Rhodococcus ruber ADH. (G) Enzymatic activity increases in mutation for 2-methylcyclohexanone in Candida parapsilosis carbonyl reductase. Download FIG S2, TIF file, 49.2 MB. Copyright © 2019 Cho et al. 2019 Cho et al. This content is distributed under the terms of the Creative Commons Attribution 4.0 International license.

10.1128/mBio.02683-18.7 TABLE S1 Sequence alignment for structure modeling. Download Table S1, DOCX file, 0.02 MB. Copyright © 2019 Cho et al. 2019 Cho et al. This content is distributed under the terms of the Creative Commons Attribution 4.0 International license.

**FIG 3:**
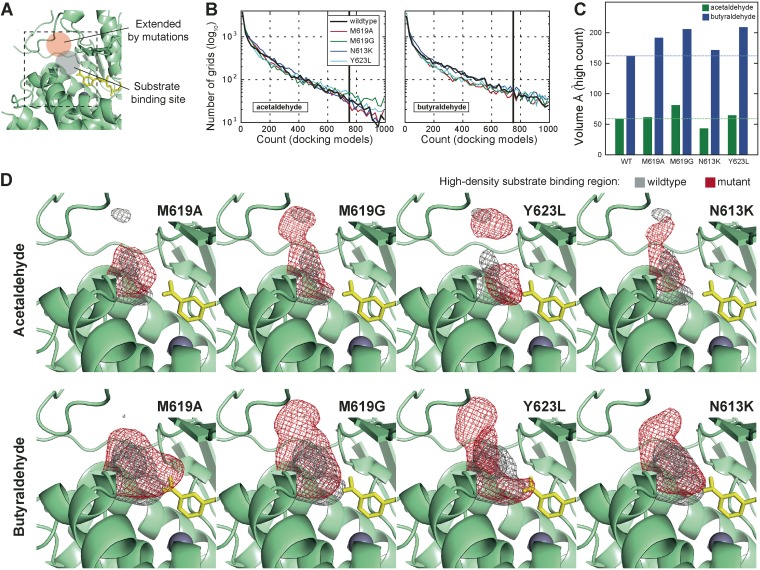
Substrate docking simulation. An acetaldehyde molecule and a butyraldehyde molecule were separately docked to the substrate binding chamber of wild-type and mutant structures. (A) Schematic view of the docking site and the substrate binding chamber region extended by mutations. (B) Distribution of voxel occupancy counts. The maximum voxel occupancy count was 1,000 (10 docking simulations × 100 maximum density [MD] snapshots). Voxels with an occupancy count above 750 were designated high-density substrate binding regions (HDR). (C) The volume of HDR for acetaldehyde and butyraldehyde. (D) Spatial distribution of HDR for each mutation.

The molecular dynamics simulations revealed that more water molecules are located at the substrate binding chambers near the active site (Fig. 2C; see also Fig. S3). This was expected for AAD_M619A_ and AAD_M619G_ as a large nonpolar amino acid at the substrate binding chamber replaced by small amino acids. Intriguingly, similar structural change was observed in AAD_Y623L_ despite the mutation site being located outside of the chamber. Still, the mutation could affect the chamber structure as it is located on the loop that forms the lining of the chamber. On the other hand, AAD_N613K_ induced only a slight change in the chamber structure although slightly greater numbers of water molecules were placed near the active site. Docking simulations performed with acetaldehyde and butyraldehyde indicated that the mutations can increase the volume of substrate binding sites (Fig. 3).

10.1128/mBio.02683-18.4 FIG S3 Change in water occupancy by mutations. Molecular dynamics simulation was employed to investigate increased (red) and decreased (blue) water density near ADHs induced by the mutations: (A) M619A. (B) M619G. (C) N613K. (D) Y623L. (E) Change in water occupancy at substrate binding chamber or water occupancy change determined by analysis within of the area within a 4-Å radius of the center of NADH O7N and N836 OE1 atoms. Download FIG S3, TIF file, 49.2 MB. Copyright © 2019 Cho et al. 2019 Cho et al. This content is distributed under the terms of the Creative Commons Attribution 4.0 International license.

On the basis of these observations, two hypotheses could be proposed for the higher butanol production capacity enabled by these mutations. The first hypothesis is that the widened substrate binding chamber would increase hydrophobic lining of the substrate chamber to allow a disproportionally longer retention time for butyraldehyde than for acetaldehyde and would consequently increase the relative levels of butanol production. The second hypothesis is that widening of the structure near the active site would allow acetaldehyde and butyraldehyde to assume more-diverse orientations at the active site. This would reduce the enzymatic activity, but the longer aliphatic chain of butyraldehyde would be less affected, leading to the retention of activity with respect to butyraldehyde.

### Mutant AADs for increased butanol selectivity.

**FIG 4:**
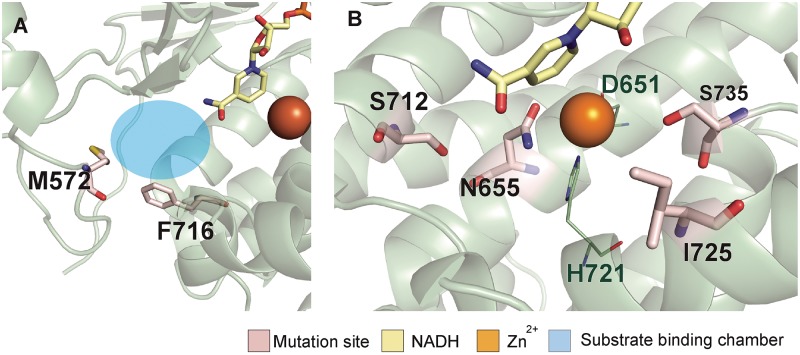
New design for increasing butanol selectivity. (A) Design for increasing the substrate binding chamber size: AAD_M572V_ and AAD_F716L_. (B) Consensus designs: AAD_M572V_, AAD_M572V_, AAD_M572V_, and AAD_M572V_ in C. acetobutylicum AAD.

On the basis of the first hypothesis, new mutations that can increase the size of the substrate binding chamber were examined. Among the amino acids located in the chamber, F716 and M572 can be replaced with smaller amino acids to enlarge the substrate binding chamber. As these residues intensively interact with the nearby atoms, only a small volume change was pursued by construction of mutants AAD_F716L_ and AAD_M572V_ (Fig. 4A). The resultant effects of these mutations, together with other those of other designed AAD mutants, are discussed below.

The second hypothesis was based on the observation that all four mutations resulted in small but consistent increases in water accessibility near the active site (Fig. 2C). Thus, it was reasoned that the slight change of structures near the active site might be responsible for the substrate selectivity. However, alternation of structures near the active site carries the potential risk of loss of enzymatic activity, as the site is highly optimized and sensitive to any variation (20). Interestingly, some active-site residues that are almost perfectly conserved in other ADHs have been replaced with other amino acids in C. acetobutylicum AAD (14, 21). As the conserved amino acids were found in many enzymatically active ADHs, it was thought that introducing them into C. acetobutylicum AAD might slightly alter its active-site structure without sacrificing its enzymatic activity. To examine this hypothesis, we constructed mutants AAD_N655H_ and AAD_S735H_, which restored the Zn^2+^ binding of histidine conserved in other ADHs, and mutants AAD_I725H_ and AAD_S712F_, which restored the conserved amino acids, whose functions have not yet been characterized clearly (Fig. 4B; see also Fig. S4).

10.1128/mBio.02683-18.5 FIG S4 Conserved sites in ADHs. (A) The sequences similar to that of C. acetobutylicum ADH were collected from the NCBI nonredundant (nr) database using BLAST (S. F. Altschul, W. Gish, W. Miller, E. W. Myers, and D. J. Lipman, 1990, J Mol Biol 215:403–410), and their profiles were displayed with WebLogo (G. E. Crooks, G. Hon, J. M. Chandonia, and S. E. Brenner, 2004, Genome Res 14:1188–1190). Residues with exceptional conservation can be found among ADHs, including the residues forming a Zn^2+^ binding site (highlighted in yellow shading). In addition, two residues near the active sites were well conserved for an unknown reason and are highlighted in blue shading. (B) The residues interacting with the Zn^2+^ ion in C. acetobutylicum AAD. (C) The active site of a typical ADH, G. thermoglucosidasius ADH (PDB ID = 3ZDR). Download FIG S4, TIF file, 53.3 MB. Copyright © 2019 Cho et al. 2019 Cho et al. This content is distributed under the terms of the Creative Commons Attribution 4.0 International license.

**FIG 5:**
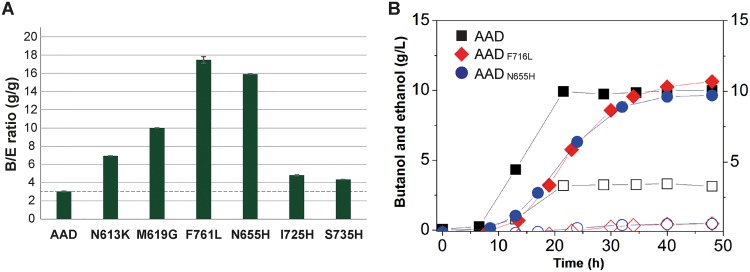
Butanol selectivity of newly designed AADs. (A) The B/E ratio of newly designed AADs. (B) Fermentation profiles of the C. acetobutylicum M5 strain expressing AAD (squares), AAD_F716L_ (diamonds), and AAD_N655H_ (circles). Closed symbols and opened symbols represent butanol production and ethanol production, respectively. Fermentations were carried out at least in duplicate for reproducibility checks, and representative profiles are presented.

Batch fermentations of the M5 mutant strains expressing these newly designed AADs were performed, and the butanol selectivities were examined (Fig. 5; see also Table S2 in the supplemental material). Interestingly, the B/E ratios were dramatically increased to 17.47 and 15.91 g/g for AAD_F716L_ and AAD_N655H_, respectively, which are 5.8-fold and 5.3-fold higher than the ratios obtained with the wild-type AAD (Fig. 5A). The main reason for the much-increased B/E ratio was the dramatic reduction in ethanol production (0.59 ± 0.01 g/liter) in the strains harboring mutant AADs. Since C. acetobutylicum strain M5 itself produces a similar amount of ethanol (0.60 ± 0.02 g/liter) without any plasmid, the level of ethanol produced by the strains expressing AAD_F716L_ and AAD_N655H_ can be considered negligible. The recombinant M5 strains expressing AAD_F716L_ and AAD_N655H_ produced 10.31 ± 0.02 and 9.71 ± 0.01 g/liter of butanol, respectively, which are amounts similar to that (10.01 ± 0.12 g/liter) produced by the control strain expressing the wild-type AAD. Thus, it was possible to achieve higher butanol selectivity by engineering the substrate binding chamber and the active site of AAD without sacrificing butanol production.

10.1128/mBio.02683-18.8 TABLE S2 Comparison of metabolites produced by newly engineered C. acetobutylicum strains. Download Table S2, DOCX file, 0.02 MB. Copyright © 2019 Cho et al. 2019 Cho et al. This content is distributed under the terms of the Creative Commons Attribution 4.0 International license.

For further validation, all of the mutant AADs were also introduced into C. acetobutylicum HKW. Similarly to what were observed with the recombinant M5 strains, the recombinant HKW strains expressing AAD_N655H_ and AAD_F716L_ showed a high level of butanol selectivity and high B/E ratios (see Table S3).

10.1128/mBio.02683-18.9 TABLE S3 Comparison of results of fermentation by newly engineered C. acetobutylicum strains. Download Table S3, DOCX file, 0.03 MB. Copyright © 2019 Cho et al. 2019 Cho et al. This content is distributed under the terms of the Creative Commons Attribution 4.0 International license.

We finally investigated whether combining these mutations would result in an even better AAD mutant (i.e., a mutant showing a higher B/E ratio). Use of the AAD_M619A-N655H_ mutation resulted in an increase in butanol production to 12.00 ± 0.20 g/liter, which was higher than the production level obtained with single mutations (see Table S3). However, ethanol production also increased, resulting in a B/E ratio of 3.97 g/g. The other combinational mutant AADs lost their functions. Simulations performed with the computational models showed a widened substrate binding chamber (see Fig. S5), but the low levels of enzymatic activities indicated that these combinatorial mutant AADs cannot form stable structures.

10.1128/mBio.02683-18.6 FIG S5 Change of water density in combinations of single mutations. The changes of water occupancy corresponding to combinatorial mutations were evaluated for highly butanol-selective mutants F716L, N655H, M619G, and N613K. The combinatorial mutations increased the volume of the substrate binding chambers, but the combinatorial effect was small. Download FIG S5, TIF file, 35.9 MB. Copyright © 2019 Cho et al. 2019 Cho et al. This content is distributed under the terms of the Creative Commons Attribution 4.0 International license.

## DISCUSSION

There have been several studies on altering butanol selectivity by metabolic engineering of clostridia (11–13). Most of the studies focused on overexpression of genes and disruption of genes to increase butanol production. For instance, strain 824 (pGROE1) overexpressing the *groESL* genes produced 17.1 g/liter of butanol and 0.97 g/liter of ethanol, resulting in a B/E ratio of 17.69 g/g (21). Also, the BEKW (pPthlD485G) strain, which was constructed by inactivating the *pta* and *buk* genes and overexpressing a mutant *adhE1* gene, produced 18.9 g/liter of butanol and 2.0 g/liter of ethanol, giving a B/E ratio of 9.45 g/g (15). C. acetobutylicum JB200, obtained through mutagenesis and adaption in a fibrous-bed bioreactor, produced 21 g/liter of butanol and 2 g/liter of ethanol, resulting in a B/E ratio of 10.5 g/g (22), while Clostridium pasteurianum MBEL_GLY2, isolated by chemical mutagenesis, was able to produce 17.3 g/liter of butanol and 0.6 g/liter of ethanol, resulting in a B/E ratio of 28.83 g/g (23). The strategies to improve butanol production in clostridia have been rather limited due to the difficulties in genetic engineering and to the limited understanding of its physiological and genetic regulation compared with other industrial microorganisms such as Escherichia coli, Bacillus subtilis, and Corynebacterium glutamicum. Much effort has been made to introduce the clostridial butanol pathway into other heterologous hosts, such as E. coli (24, 25), Saccharomyces cerevisiae (26), Lactobacillus brevis (27), Pseudomonas putida (28), and B. subtilis (28). Among these microorganisms, engineered E. coli has been the best studied. Starting with introduction of the butanol pathway into E. coli (24, 25), pathway optimization studies were performed to increase butanol production (29, 30). In a recent study, E. coli was systematically engineered to produce 20 g/liter of butanol and 0.5 g/liter of ethanol, with results showing a high B/E ratio of ∼40 g/g in the absence of vectors, antibiotics, and inducers (31). To reach this goal, 33 native genes related to by-products forming pathways (ethanol, lactate, acetate, and succinate) were deleted and 5 heterologous genes related to the butanol pathway were introduced. Furthermore, adaptive evolution and Tn*5* transposon mutation were conducted (31). This great work did not consider enzyme engineering studies to change substrate specificities related to butanol production. Incorporation of enzyme engineering results from our study in engineering E. coli might result in further increases in the B/E ratio. On the other hand, such large-scale metabolic engineering performed in E. coli as described above is still quite difficult (although doable to some extent) in C. acetobutylicum. In the future, it is expected that combining systems metabolic engineering and enzyme engineering in clostridia will result in production of butanol to a high titer with high yield, productivity, and butanol selectivity.

AAD encoded by the *adhE1* gene is the key enzyme in butanol production by C. acetobutylicum but is also directly involved in ethanol production. In this study, the substrate binding chamber and the active site of AAD were engineered to modulate the butanol and ethanol selectivities of AAD. The resultant enzymes showed remarkably increased *in vivo* selectivity of butanol versus ethanol. The high butanol selectivity of the newly engineered enzyme was due to the drastic reduction in ethanol production (Fig. 5B). The butanol selectivity can be affected by interactions of AAD with butyraldehyde and acetaldehyde, since the binding of butyraldehyde to AAD inhibits the binding of acetaldehyde. The increased butanol selectivity could be explained by reduced activity of ethanol dehydrogenase (EDH) or by a longer retention time for butyraldehyde than acetaldehyde (Table 2). Thus, it would be important to consider both enzyme-substrate interactions and enzyme-mediated substrate-substrate interactions in designing an enzyme with altered selectivity.

**TABLE 2:** EDH and BDH enzyme activity test in the recombinant strains

	Specific activity^*a*^ (mU/mg protein)	
Strain	EDH	BDH
M5 (pTHL1-AdhE1)	6.13 ± 0.25	12.77 ± 2.13
M5 (pTHL1-M619G)	7.21 ± 2.03	37.40 ± 5.25
M5 (pTHL1-N655H)	2.66 ± 1.55	5.01 ± 0.38
M5 (pTHL1-F716L)	3.13 ± 1.39	5.66 ± 0.93

a All results shown represent means ± standard deviations of results from triplicate experiments.

We further examined ineffective designs and found some clues about AAD functionality and for further improved design. AAD_S712F_ and AAD_M572V_ significantly reduced butanol production, suggesting structural instability (see Table S2 in the supplemental material). AAD_S712F_ was designed to restore the conserved amino acid present in other ADHs, but its loss of butanol production suggested that C. acetobutylicum AAD might have been differently evolved to optimize butanol production. Thus, the S712 residue seems to have an important role in butanol production in C. acetobutylicum. Both AAD_S735H_ and AAD_N655H_ were expected to restore the conserved histidine–Zn^2+^ ion interaction, but only AAD_N655H_ significantly increased butanol selectivity. This result can be explained by the different locations of the two mutants, where N655 is located on the substrate binding side whereas S735 is placed on the NADH binding site. AAD_I725H_ restores the conserved amino acid but has shown little effect on substrate selectivity.

Among various aldehyde/alcohol dehydrogenases in C. acetobutylicum, we focused on AAD, which is well known as the key enzyme for butanol production (13). Interestingly, AdhE2, encoded by the *adhE2* gene located on the pSOL1 megaplasmid, showed 66% amino acid sequence identity to AAD. The overall structure model of AdhE2 is quite similar to that of AAD, but its active-site structure is similar to those of conventional alcohol dehydrogenases (see Fig. C in Text S1 in the supplemental material). Although the *adhE2* gene is expressed under alcohologenic conditions for butanol production (32, 33), its structural similarity to AAD suggests that AdhE2 can also be utilized as a new platform for similar mutational study.

In this study, only biochemical data were used to characterize the newly designed mutations. In future work, however, structural studies, including cocrystallization with substrates, will be needed for detailed understanding of the mechanisms. Eventually, combinational optimization of the aldehyde/alcohol dehydrogenases and also further metabolic engineering can be performed to ultimately achieve sole fermentation of butanol in C. acetobutylicum.

In conclusion, we developed mutant AADs that can selectively produce butanol by combining the strategies of random mutagenesis, substrate selectivity screening, and computational structural analysis. We demonstrated that substrate selectivity can be increased by directly engineering the enzyme. We expect that the combination of experimental and rational design approaches suggested here can be utilized for engineering other enzymes to increase product selectivity and, consequently, to develop more efficient engineered strains capable of producing valuable chemicals and fuels from renewable resources.

## MATERIALS AND METHODS

### Bacterial strains and culture condition.

The bacterial strains used in this study are listed in Table A in Text S1 in the supplemental material. C. acetobutylicum cells were cultured anaerobically at 37°C in clostridial growth medium (CGM), which contains the following (per liter): 0.75 g KH_2_PO_4_, 0.75 g K_2_HPO_4_, 1.0 g NaCl, 0.017 g MnSO_4_ · 5H_2_O, 0.70 g MgSO_4_ · 7H_2_O, 0.01 g FeSO_4_ · 7H_2_O, 2.0 g l-asparagine, 5.0 g yeast extract, 2.0 g (NH_4_)_2_SO_4_, and 80 g glucose. For growth on solid medium, cells were cultured anaerobically at 37°C on 2× YTG (pH 5.8) (16 g Bacto tryptone, 10 g yeast extract, 4 g NaCl, 5 g glucose per liter) agar plates. For the screening of transformants, erythromycin and thiamphenicol were used at final concentrations of 40 mg/liter and 5 mg/liter, respectively.

### Plasmid construction and transformation.

The plasmids and primers used in this study are listed in Table A in Text S1. Restriction enzymes were purchased from Enzynomics (Daejeon, Republic of Korea) and New England Biolabs (Ipswich, MA, USA). T4 DNA ligase (Elpisbio, Daejeon, Republic of Korea) was used for ligation, and proofreading *Pfu* DNA polymerase (Solgent, Daejeon, Republic of Korea) was used for PCR.

The C. acetobutylicum *adhE1* gene was amplified by PCR with primers AdhE1-F and AdhE1-R using genomic DNA of C. acetobutylicum ATCC 824 as a template. The resulting fragment and pTHL1-Cm were digested with PstI and AvaI and were ligated to make pTHL1-AdhE1.

The amino acid residues at positions 613, 619, and 623 (Asn, Met, and Tyr of C. acetobutylicum AAD) were determined by alignment of C. acetobutylicum AAD and Z. mobilis ADH2 (Fig. 1). To construct the corresponding mutant library, two DNA fragments of the *adhE1* gene were amplified using the genomic DNA of C. acetobutylicum ATCC 824 and primer pairs AdhE1-F/N613-R and N613-F/AdhE1-R. Overlapping PCR was performed with a mixture of the two amplified products using primers AdhE1-F and AdhE1-R. The PCR products were digested with PstI and AvaI and ligated with PstI- and AvaI-treated pTHL1-Cm to construct mutant libraries. The mutant libraries for M619 and N623 were constructed in the same manner. Methylated libraries were transformed into strain HKW by electroporation (Bio-Rad, Hercules, CA, USA) (2.5 kV, ∞ Ω, and 25 μF).

### Screening of library.

Single colonies were picked up from the agar plates and inoculated into a 24-well microtiter plate (SPL, Gyeonggi-do, Republic of Korea) containing 2 ml of CGM supplemented with 5 mg/liter thiamphenicol as the first screening (see Fig. A in Text S1). After 48 h of incubation in the anaerobic chamber, culture broths were taken out to analyze the concentration of metabolites using a gas chromatography system (model 7890; Agilent Technologies, CA, USA) equipped with an 80/120 Carbopack B AW packed glass column (Supelco, PA, USA) and a flame ionization detector (FID; Agilent Technologies, CA, USA). The second screening was performed in a serum bottle containing 30 ml of CGM with 5 mg/liter thiamphenicol. After 48 h of cultivation at 37°C, culture broths were taken out to analyze the production of metabolites using gas chromatography.

### Batch fermentation.

Batch fermentation was performed in a 5.0-liter LiFlus GX bioreactor (Biotron, Kyunggi-Do, Republic of Korea) containing 1.8 liters of CGM supplemented with 80 g/liter glucose. To prepare a preculture, a single colony was inoculated into a test tube containing 10 ml CGM supplemented with 80 g/liter glucose. This preculture was transferred into a 500-ml flask containing 200 ml CGM supplemented with 80 g/liter glucose until the cell density reached an optical density at 600 nm (OD_600_) of 2.0. After that, cells were inoculated into the bioreactor. The pH was adjusted to 5.5 with 14% (vol/vol) ammonia solution and was not controlled when it a level above 5.5 during the fermentation. The bioreactor was operated at 37°C and 200 rpm, and oxygen-free nitrogen gas was supplied at a flow rate of 0.5 liters/min after passage through an oxygen trap (Agilent, Santa Clara, CA, USA).

### Analytical techniques.

Cell growth was monitored by measuring the optical density at 600 nm (OD_600_) using an Ultraspec 3100 Pro spectrophotometer (Amersham Biosciences, Uppsala, Sweden). The relative concentrations of solvent and butanol were analyzed by the use of a gas chromatograph (model 7890; Agilent Technologies, CA, USA) equipped with an 80/120 Carbopack B AW packed glass column (Supelco, PA, USA) and a flame ionization detector (FID; Agilent Technologies, CA, USA). The concentrations of glucose and organic acids were assessed with using a high-performance liquid chromatograph (Waters 1515/2414/2707; Waters, MA, USA). A MetaCarb 87H column (Agilent Technologies, CA, USA) (300 by 7.8 mm) was eluted isocratically with 0.01 N H_2_SO_4_ at 35°C at a flow rate of 0.5 ml/min.

### ADH structure modeling.

The proteins evolutionally related to C. acetobutylicum AAD were retrieved from the NCBI nonredundant database (34) using BLAST (35), and the G. thermoglucosidasius ADH (PDB ID = 3ZDR) showed the highest (48%) sequence similarity to the C. acetobutylicum ADH. The structure of C. acetobutylicum ADH was modeled using MODELLER 9v11 (36) on the basis of the G. thermoglucosidasius ADH structure.

### Molecular dynamics simulation.

The structure models of C. acetobutylicum AAD and its variants were subjected to molecular dynamics simulation using the GROMACS 4.5.1 suite (37). An Amber03 force field was used, and the TIP3P water model was used. Water molecules were placed into the analysis box (with the box boundary 1.0 nm distant from any protein atoms), and the system was neutralized by adding counter ions (Cl^-^). The system was stabilized with 5,000 steps of steep-descent energy minimization followed by 50 ps of equilibration simulation under protein heavy-atom position constraints. A total of 5 ns of main simulation was performed at 298 K using a Berendsen thermostat and LINCS algorithm constraint. For each structure model, five independent simulations were executed to reduce simulation bias. The parameters used in the simulation can be found in Table B in Text S1.

### Water occupancy analysis.

Water occupancies around AADs were calculated for the wild types and mutants, and their differences were evaluated. To calculate water occupancy, 5,000 structure snapshots were retrieved from each simulation trajectory, and their protein parts were superimposed. The coordinates of water molecules were transformed accordingly. Three-dimensional grids with 0.7-Å intervals were applied around the protein molecule, and the grids within a 1.4-Å radius of water oxygen atoms were assumed to be occupied by the water molecules. The grid occupancy data were averaged for all snapshots to calculate water occupancy fields.

### Site-directed mutagenesis.

The modified site-directed mutagenesis protocol was applied to construct the mutants (38). All primers used here are listed in Table A in Text S1. To construct the pTHL1-M572V plasmid, PCR amplification was performed using pTHL1-AdhE1 as the template and primers M572V-F and M572V-R. A 5-μl volume of the purified PCR product was directly transformed into TOP10 competent cells. The transformant was plated on Luria-Bertani (LB) agar plates (1.5% [wt/vol]) supplemented with 50 mg/liter ampicillin. The N655H, S712F, F716L, I725H, and S735H mutants were constructed in the same manner.

### Assay of alcohol dehydrogenase activity.

To measure ethanol dehydrogenase (EDH) and butanol dehydrogenase (BDH) activities, cells cultured in static flasks were harvested at an OD_600_ of 1.0 and washed with 10 ml lysis buffer (0.1 mM dithiothreitol [DTT] and 0.1 M Tris-HCl, pH 7.6). Pellets were resuspended with 3 ml lysis buffer, anaerobically lysed by the use of a Bioruptor sonicator (Diagenode, NJ, USA), and centrifuged at 14,000 × *g* for 25 min at 4°C. After measurement of total protein concentrations by the Bradford method, the assay was performed with a GeneQuant 1300 spectrophotometer (GE Healthcare, IL, USA), monitoring decreases in absorbance at 340 nm. The reaction mixture contained 0.1 M Tris-HCl (pH 7.6), 5 mM DTT, 300 μM NADH, and 50 mM butyraldehyde or 150 mM acetaldehyde. The reaction was initiated by the addition of 100 μl of extract to 1 ml of reaction mixture. One unit of alcohol dehydrogenase activity was defined as the amount of enzyme necessary for the consumption of 1 μmol NADH per min. All experiments were carried out in at least triplicate under anaerobic conditions.

### Nucleotide sequence accession numbers.

The nucleotide sequences of mutant AADs have been deposited at GenBank under the following accession numbers: MK307997 to MK308006.
