# Supplementary material for: Engineering Clostridial Aldehyde/Alcohol Dehydrogenase for Selective Butanol Production
Source: mBio. 2019 Jan 22;10(1):e02683-18. doi: 10.1128/mBio.02683-18 (PMC6343042; doi:10.1128/mBio.02683-18)
Supplement: TEXT S1 [file mBio.02683-18-s0001.doc]

TEXT S1. Supplemental Figures and Tables

Engineering clostridial aldehyde/alcohol dehydrogenase AAD for selective butanol production

Changhee Cho, Seungpyo Hong, Hyeon Gi Moon, Yu-Sin Jang, Dongsup Kim,
and Sang Yup Lee


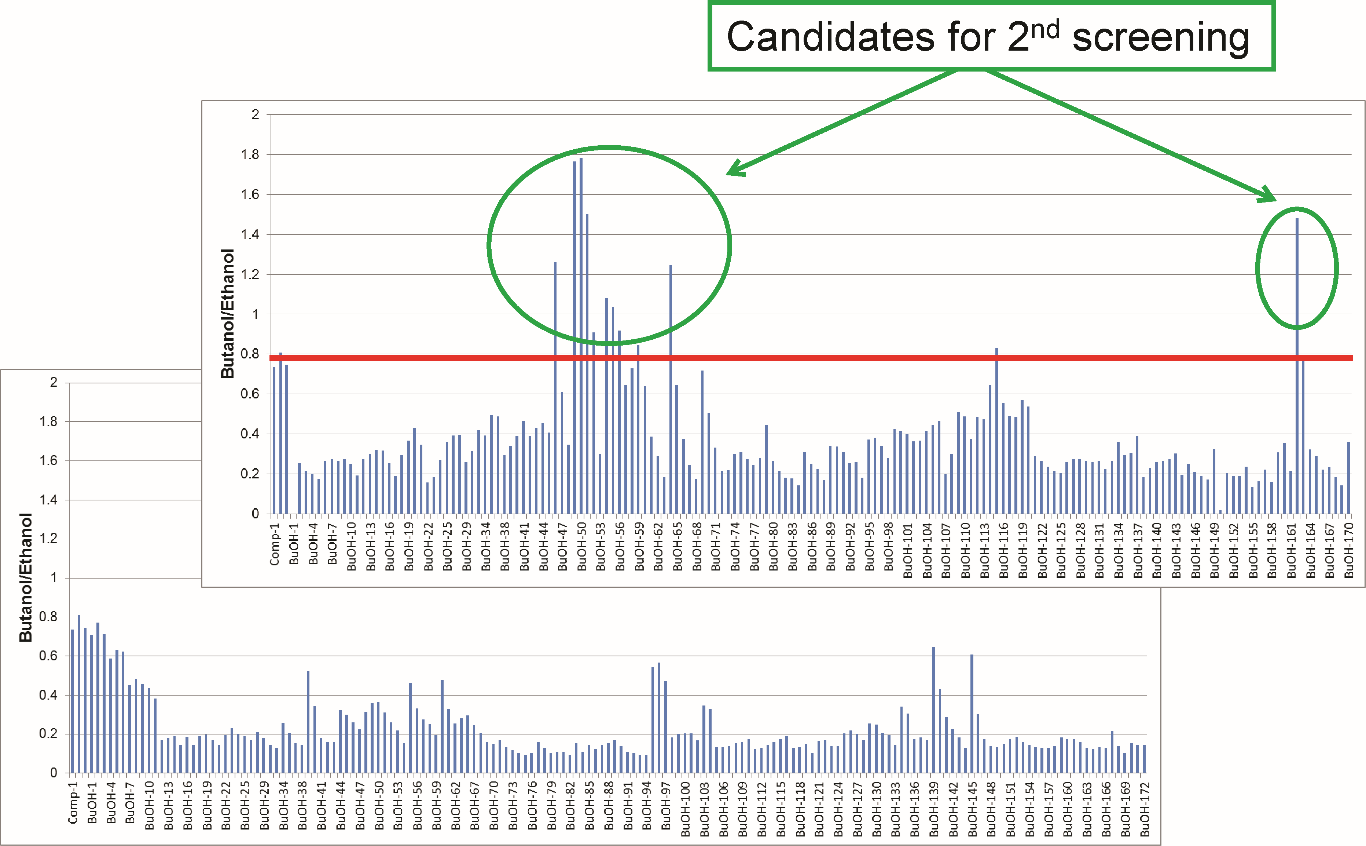


**FIG A** The first screening of mutant libraries. Production of butanol and ethanol was analyzed by gas chromatography and the ratio of butanol to ethanol was indicated. The red line means the ratio of butanol to ethanol of HKW (pTHL1-AdhE1) strain as a positive control. The strains shown in X-axis are: Comp-1, HKW (pTHL1-AdhE1); BuOH-number, each transformant.


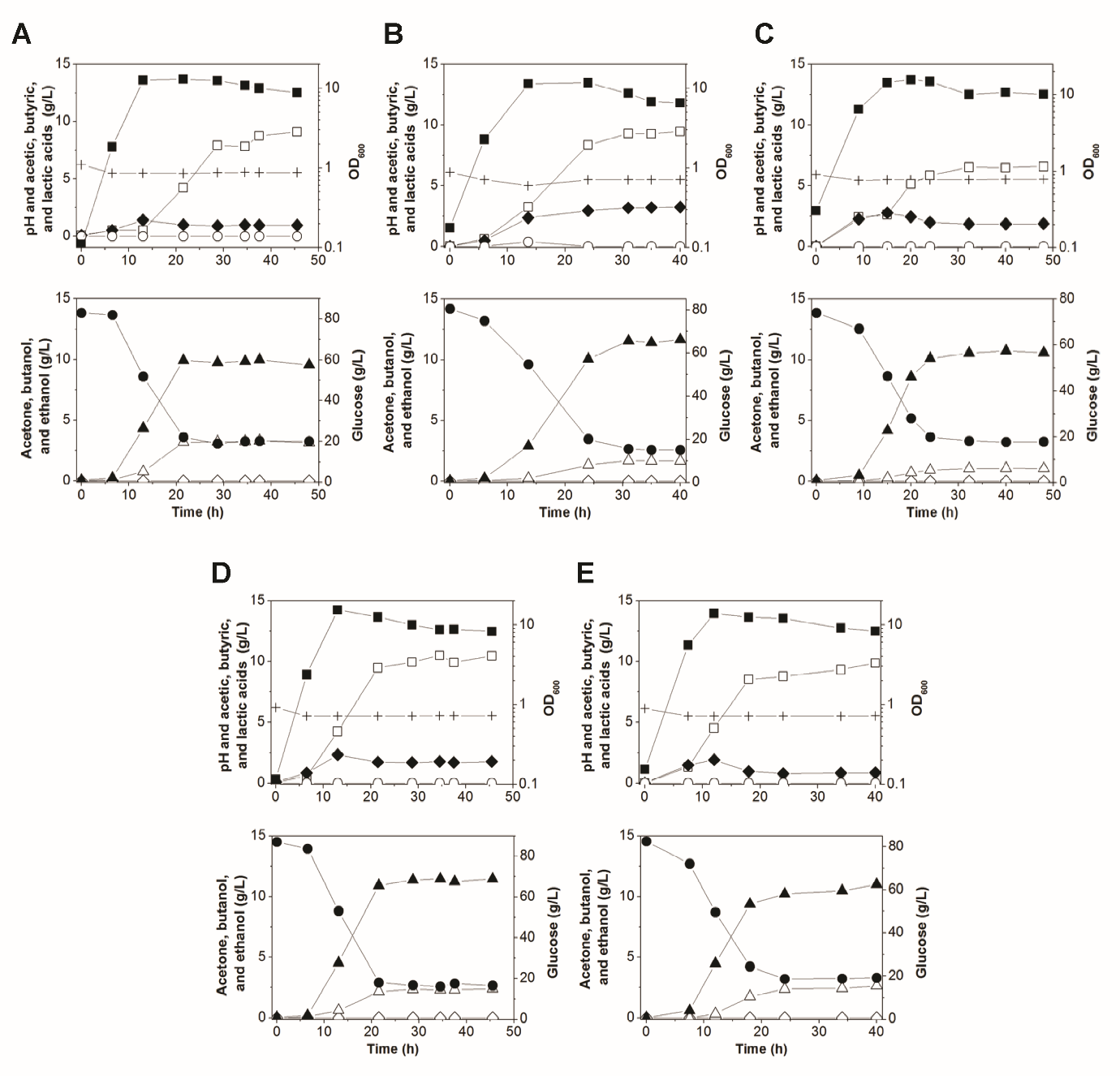


**FIG B** Time profiles of batch fermentation. (A) *C. acetobutylicum* M5 (pTHL1-AdhE1), (B) M5 (pTHL1-N613K), (C) M5 (pTHL1-M619A), (D) M5 (pTHL1-M619G), and (E) M5 (pTHL1-Y623L). Symbols are: ■, OD_600_; □, acetic acid; ○, lactic acid; ◆, butyric acid; +, pH; ●, glucose; ◇, acetone; △, ethanol; ▲, butanol. Fermentations were carried out at least in duplicate for reproducibility check, and representative profiles are presented.


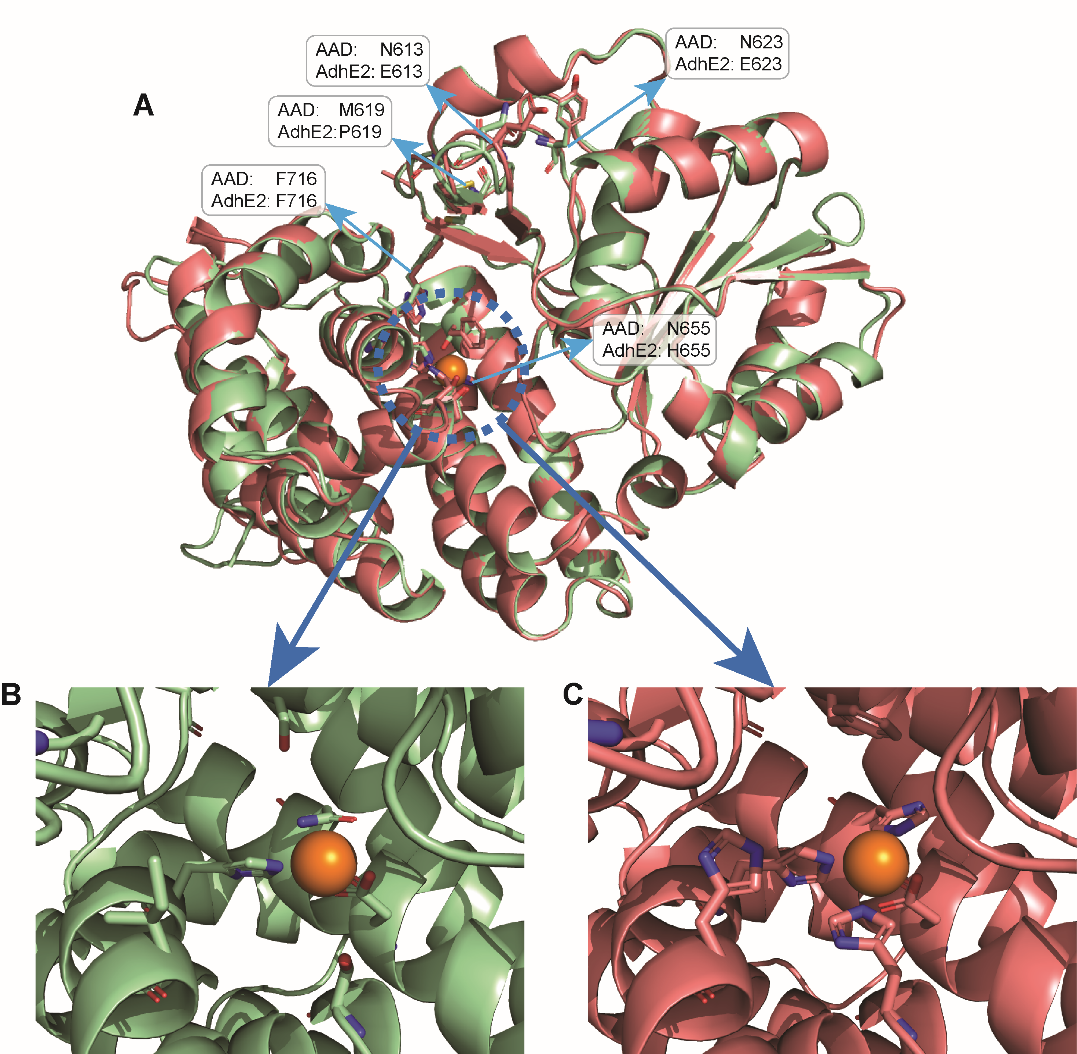


**FIG C** Structural models of *C. acetobutylicum* AAD and AdhE2. (A) Comparison of *C. acetobutylicum* AAD (green) with AdhE2 (red). Key mutation sites and their amino acids were marked in the models. (B) Near active site of *C. acetobutylicum* AAD. (C) Near active site of AdhE2.

**Table A** Bacterial strains and plasmids used in this study

| Strain | Relevant characteristics*^a^* | Source |
| --- | --- | --- |
| *C. acetobutylicum* |  |  |
| HKW | *adhE1::intron,adhE1-mutant of C. acetobutylicum wild-type strain (ATCC 824)* | This study |
| HKW (pTHL1-AdhE1) | *C. acetobutylicum* HKW harboring pTHL1-AdhE1 |  |
| M5 | pSOL- |  |
| M5 (pTHL1-AdhE1) | *C. acetobutylicum* M5 harboring pTHL1-AdhE1 | This study |
| M5 (pTHL1-N613K) | *C. acetobutylicum* M5 harboring pTHL1-N613K | This study |
| M5 (pTHL1-M619A) | *C. acetobutylicum* M5 harboring pTHL1-M619A | This study |
| M5 (pTHL1-M619G) | *C. acetobutylicum* M5 harboring pTHL1-M619G | This study |
| M5 (pTHL1-Y623L) | *C. acetobutylicum* M5 harboring pTHL1-M623L | This study |
| M5 (pTHL1-M572V) | *C. acetobutylicum* M5 harboring pTHL1-M572V | This study |
| M5 (pTHL1-N655H) | *C. acetobutylicum* M5 harboring pTHL1-N655H | This study |
| M5 (pTHL1-S712F) | *C. acetobutylicum* M5 harboring pTHL1-S712F | This study |
| M5 (pTHL1-F716L) | *C. acetobutylicum* M5 harboring pTHL1-F716L | This study |
| M5 (pTHL1-I725H) | *C. acetobutylicum* M5 harboring pTHL1-I725H | This study |
| M5 (pTHL1-S735H) | *C. acetobutylicum* M5 harboring pTHL1-S735H | This study |
|  |  |  |
| *E. coli* |  |  |
| Top10 | Host for cloning | Invitrogen*^b^* |
| ER2925(pAN1) | ER2925 harboring pAN1 | This study |
| Plasmids | Relevant characteristics | Source |
| pAN1 | Cm^r^ Tc^r^ Φ3T I |  |
| pTHL1-Cm | Ap^r^ Em^r^ Cm^r^ P*_thl_ C. acetobutylicum* expression vector |  |
| pTHL1-AdhE1 | Ap^r^ Em^r^ Cm^r^ P*_thl_::adhE1* | This study |
| pTHL1-MU613 | Ap^r^ Em^r^ Cm^r^ P*_thl_::adhE1-613 position randomly replaced* | This study |
| pTHL1-MU619 | Ap^r^ Em^r^ Cm^r^ P*_thl_::adhE1-619 position randomly replaced* | This study |
| pTHL1-MU623 | Ap^r^ Em^r^ Cm^r^ P*_thl_::adhE1-623 position randomly replaced* | This study |
| pTHL1-N613K | Ap^r^ Em^r^ Cm^r^ P*_thl_::adhE1^N613K^* | This study |
| pTHL1-M619A | Ap^r^ Em^r^ Cm^r^ P*_thl_::adhE1^M619A^* | This study |
| pTHL1-M619G | Ap^r^ Em^r^ Cm^r^ P*_thl_::adhE1^M619G^* | This study |
| pTHL1-Y623L | Ap^r^ Em^r^ Cm^r^ P*_thl_::adhE1^Y623L^* | This study |
| pTHL1-M572V | Ap^r^ Em^r^ Cm^r^ P*_thl_::adhE1^M572V^* | This study |
| pTHL1-N655H | Ap^r^ Em^r^ Cm^r^ P*_thl_::adhE1^N655H^* | This study |
| pTHL1-S712F | Ap^r^ Em^r^ Cm^r^ P*_thl_::adhE1^S712F^* | This study |
| pTHL1-F716L | Ap^r^ Em^r^ Cm^r^ P*_thl_::adhE1^F716L^* | This study |
| pTHL1-I725H | Ap^r^ Em^r^ Cm^r^ P*_thl_::adhE1^I725H^* | This study |
| pTHL1-S735H | Ap^r^ Em^r^ Cm^r^ P*_thl_::adhE1^S735H^* | This study |
| Primers | Sequence (5’- 3’) | Source |
| AdhE1-F | AAAACTGCAGTTTATGAAAGTCACAACAGTAAAGG | This study |
| AdhE1-R | CCCCCCGGGGGGGTTGAAATATGAAGGTTTAAGGTTG | This study |
| N613-R | GCTAACATGTACTTATTTCCAGTNNNATTGTCAGTTACTAAAGCAAAAG | This study |
| N613-F | CTTTTGCTTTAGTAACTGACAATNNNACTGGAAATAAGTACATGTTAGC | This study |
| M619-R | GTCATTTCATAATCTGCTAANNNGTACTTATTTCCAGTGTTATTG | This study |
| M619-F | CAATAACACTGGAAATAAGTACNNNTTAGCAGATTATGAAATGAC | This study |
| N623-R | GCCATATTTGGTGTCATTTCNNNATCTGCTAACATGTACTTATTTC | This study |
| N623-F | GAAATAAGTACATGTTAGCAGATNNNGAAATGACACCAAATATGGC | This study |
| M572V-F | CTTGCAATAAAATTTGTAGACATAAGAAAG | This study |
| M572V-R | CTTTCTTATGTCTACAAATTTTATTGCAAG | This study |
| N655H-F | TAGATGCACTAGTACACAGTATAGAAGCAT | This study |
| N655H-R | ATGCTTCTATACTGTGTACTAGTGCATCTA | This study |
| S712F-F | ATGGCAGGTATGGCATTTGCTAATGCATTT | This study |
| S712F-R | AAATGCATTAGCAAATGCCATACCTGCCAT | This study |
| F716L-F | TCCGCTAATGCATTACTAGGTCTATGTCAT | This study |
| F716L-R | ATGACATAGACCTAGTAATGCATTAGCGGA | This study |
| I725H-F | GTCATTCCATGGCACATAAATTAAGTTCAG | This study |
| I725H-R | CTGAACTTAATTTATGTGCCATGGAATGAC | This study |
| S735H-F | AACACAATATTCCTCATGGCATTGCCAATG | This study |
| S735H-R | CATTGGCAATGCCATGAGGAATATTGTGTT | This study |

*^a^* Cm^r^, chloramphenicol/thiamphenicol resistance gene; Em^r^, erythromycin-resistance gene; Ap^r^, ampicillin-resistance gene

*^b^* Invitrogen, Invitrogen Corporation, Carlsbad, Ca, USA

**SI References**

1. **Clark SW, Bennett GN, Rudolph FB.** 1989. Isolation and characterization of mutants of *Clostridium acetobutylicum* ATCC 824 deficient in acetoacetyl-coenzyme A:acetate/butyrate:boenzyme A-transferase (EC 2.8.3.9) and in other solvent pathway enzymes. Appl. Environ. Microbiol. 55:970-976.

2. **Mermelstein LD, Papoutsakis ET.** 1993. *In vivo* methylation in *Escherichia coli* by the *Bacillus subtilis* phage phi 3T I methyltransferase to protect plasmids from restriction upon transformation of *Clostridium acetobutylicum* ATCC 824. Appl. Environ. Microbiol. 59:1077-1081.

3. **Jang YS, Malaviya A, Lee J, Im JA, Lee SY, Eom MH, Cho JH, Seung D Y.** 2013. Metabolic engineering of *Clostridium acetobutylicum* for the enhanced production of isopropanol-butanol-ethanol fuel mixture. Biotechnol. Prog. 29:1083-1088.

**Table B** Molecular dynamics simulation parameters

| **Parameters** | **Values** |
| --- | --- |
| **Simulation suite** | GROMACS 4.5.1 |
| **Force field** | AMBER03 |
| **Water model** | TIP3P |
| **Coulomb type** | PME |
| **VDW type** | Cut-off |
| **VDW cutoff** | 1 nm |
| **Reference temperature** | 298 K |
| **Temperature coupling** | Berendsen |
| **Pressure coupling** | Berendsen |
| **Pressure coupling type** | Isotropic |
| **Constraints** | All-bonds |
| **Constraint algorithm** | LINCS |
| **Time step** | 2 fs |
| **Number of steps** | 2,500,000 |
| **Structure sampling interval** | 1000 |
