# Supplementary figures and images for: Engineering Clostridial Aldehyde/Alcohol Dehydrogenase for Selective Butanol Production
Source: mBio. 2019 Jan 22;10(1):e02683-18. doi: 10.1128/mBio.02683-18 (PMC6343042; doi:10.1128/mBio.02683-18)

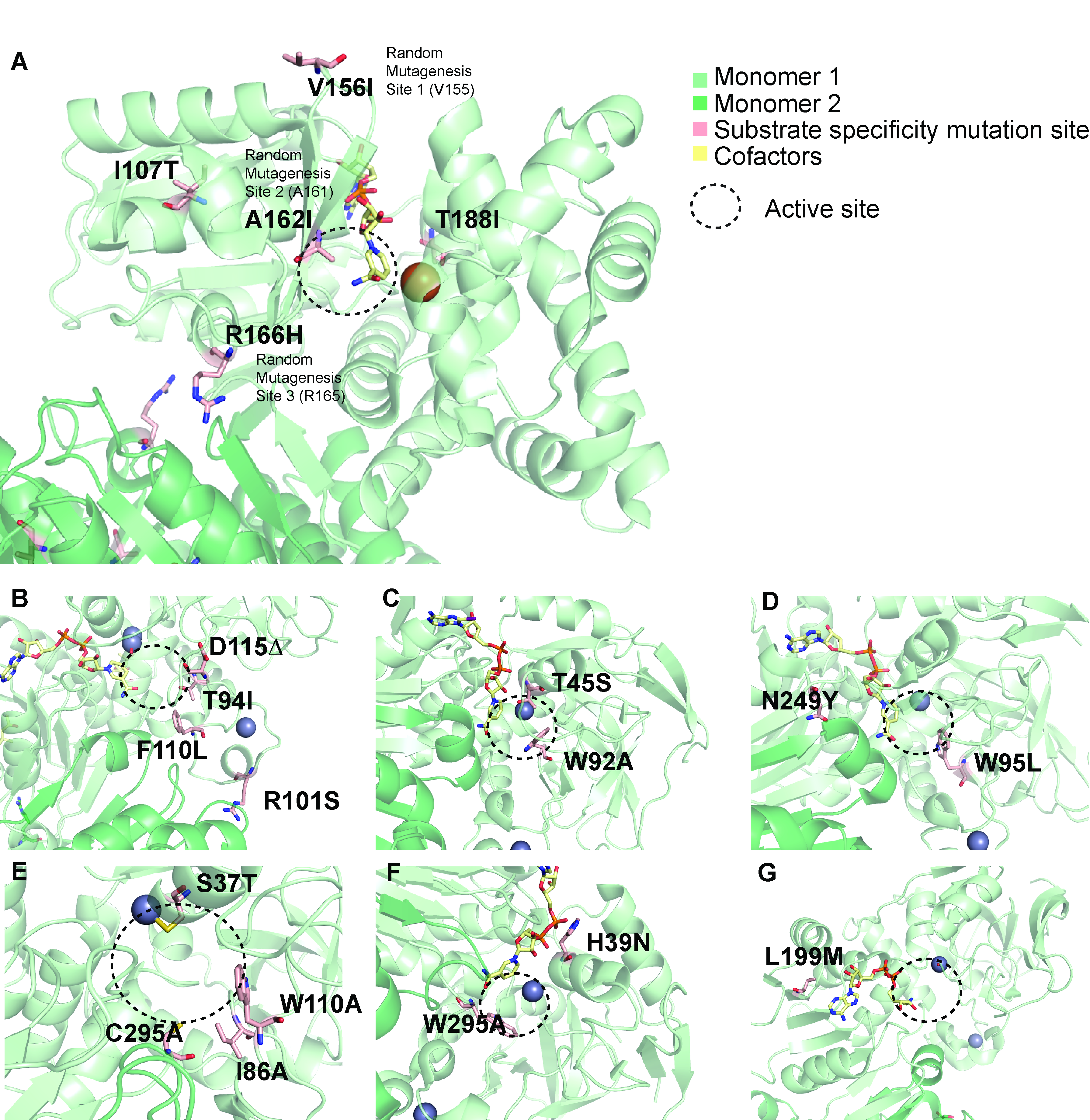

Supplement: FIG S2 [file mBio.02683-18-sf002.tif]

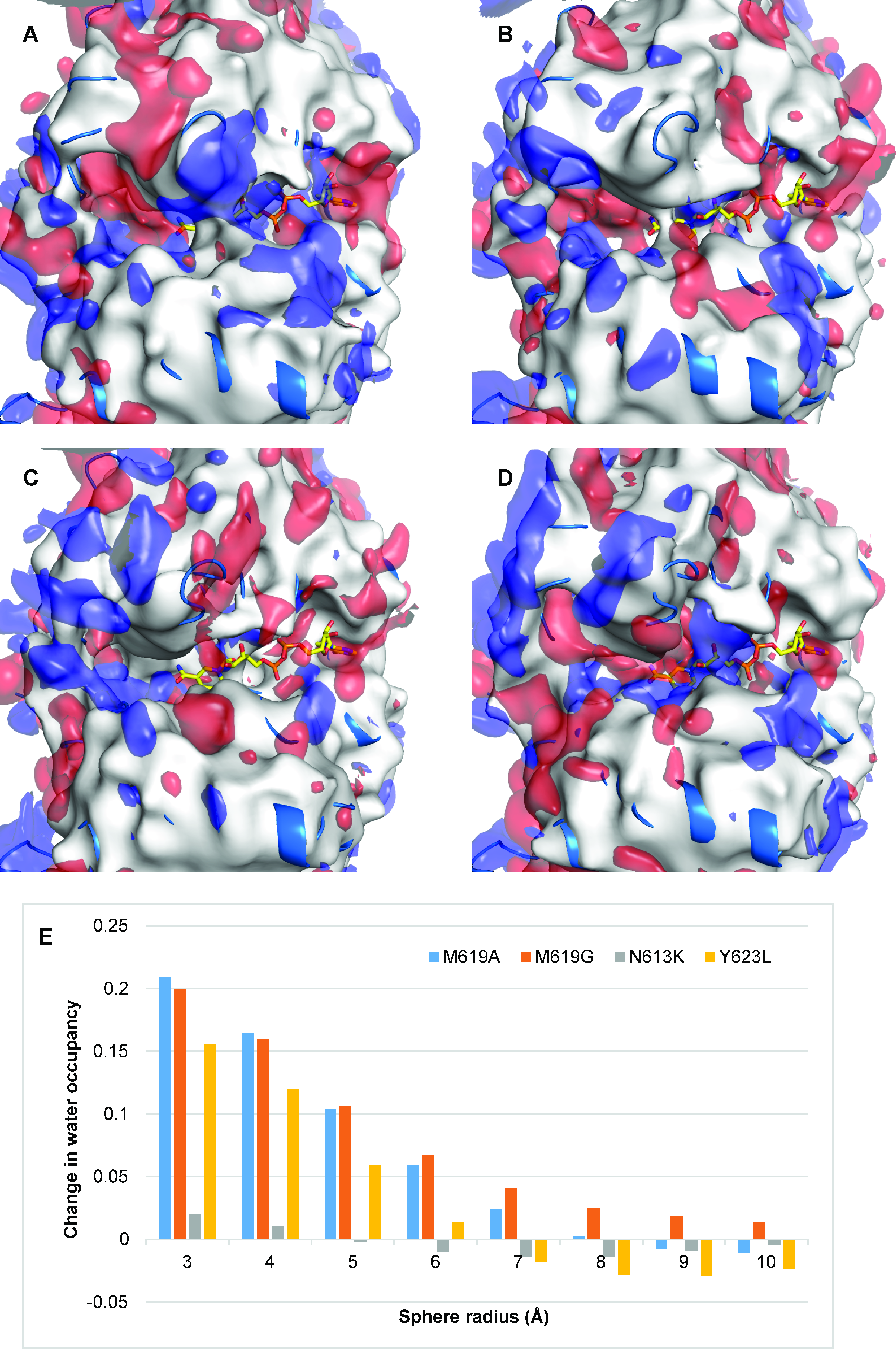

Supplement: FIG S3 [file mBio.02683-18-sf003.tif]

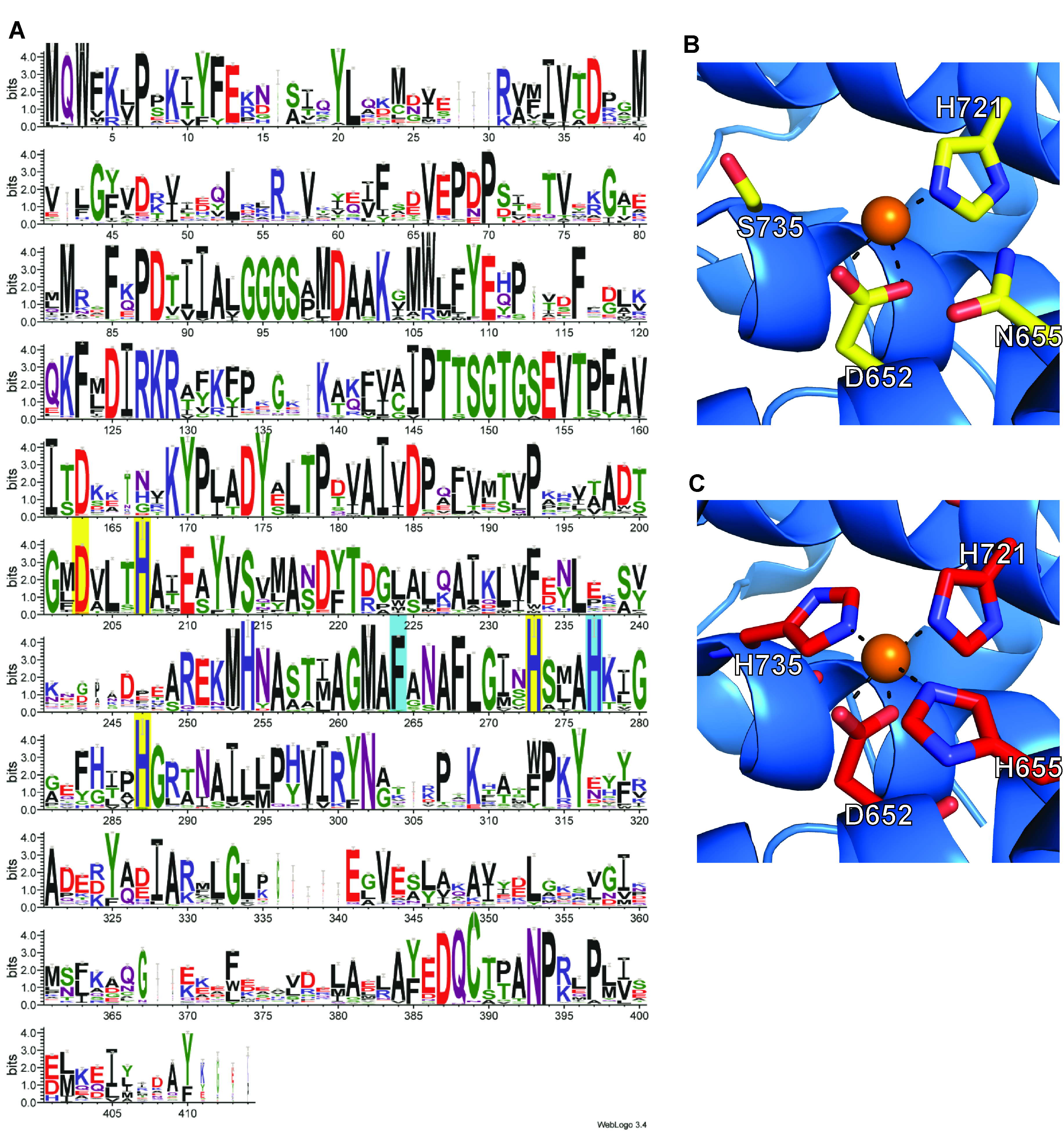

Supplement: FIG S4 [file mBio.02683-18-sf004.tif]

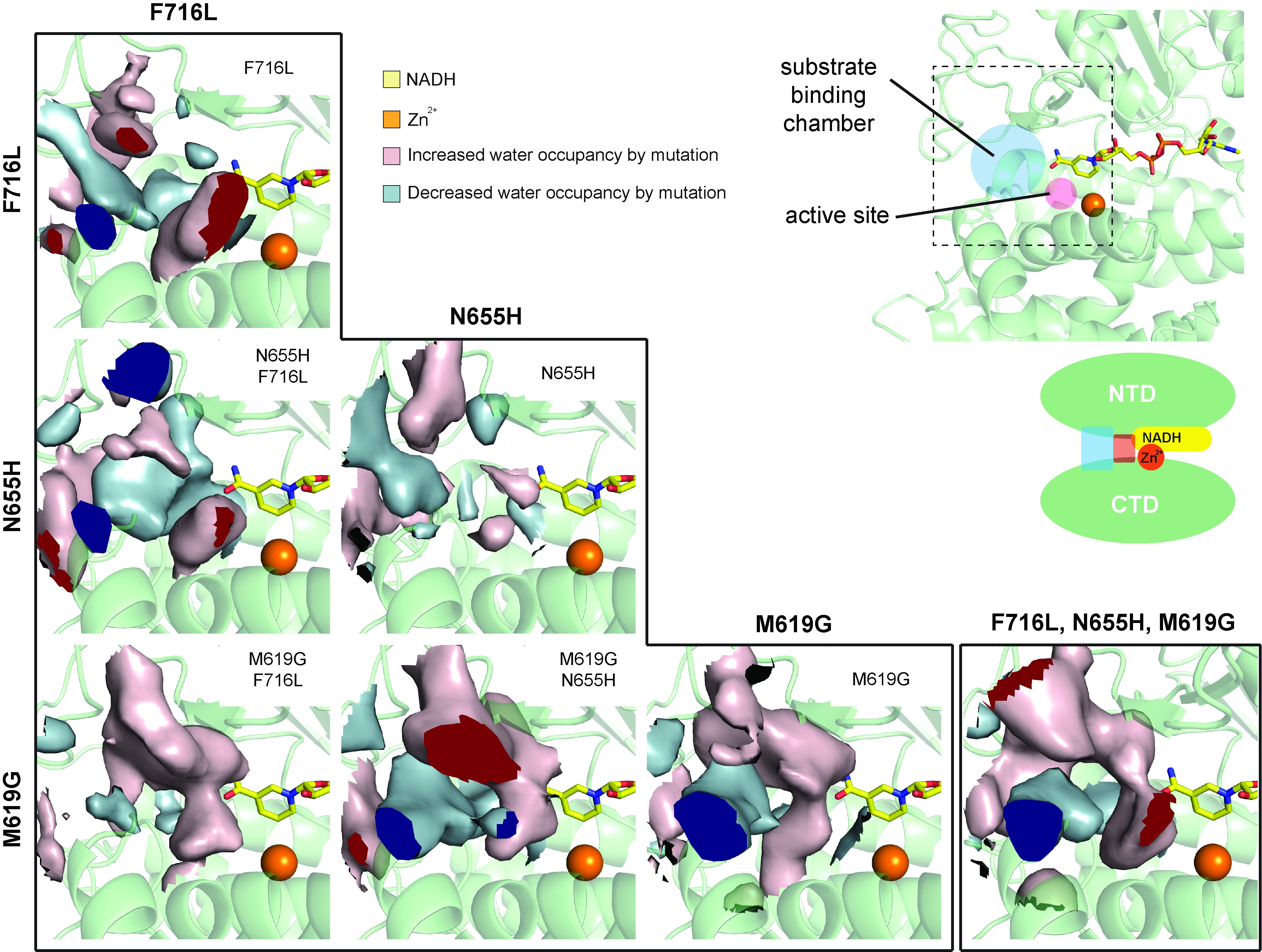

Supplement: FIG S5 [file mBio.02683-18-sf005.tif]
