# Supplementary material for: Engineering Clostridial Aldehyde/Alcohol Dehydrogenase for Selective Butanol Production
Source: mBio. 2019 Jan 22;10(1):e02683-18. doi: 10.1128/mBio.02683-18 (PMC6343042; doi:10.1128/mBio.02683-18)
Supplement: TABLE S1 [file mBio.02683-18-st001.docx]

**Table S1** Sequence alignment for structure modeling

|  | Alignment |
| --- | --- |
| Template^a^ | NMQWFKVPPKIYFEKN-AVQYLAKMPDIS---RAFIVTDP |
| Sequence^b^ | -MLWFRVPHKVYFKFGCLQFALKDL-KDLKKKRAFIVTDS |
| Template | GMV-KLGYVDKVLYYLRRRPDYVHSEIFSEVEPDPSIETV |
| Sequence | DPYN-LNYVDSIIKILEHLD--IDFKVFNKVGREADLKTI |
| Template | MKGVDMMRSFEPDVIIALGGGSPMDAAKAMWLFYEHPT-A |
| Sequence | KKATEEMSSFMPDTIIALGGTPEMSSAKLMWVLYEHP-EV |
| Template | DFNALKQKFLDIRKRVYKYPKLGQ-KAKFVAIPTTSGTGS |
| Sequence | KFEDLAIKFMDIRKRIYTFPK-LGKKAMLVAITTSAGSGS |
| Template | EVTSFAVITDKK-TNIKYPLADYELTPDVAIVDPQFVMTV |
| Sequence | EVTPFALVTDNNT-GNKYMLADYEMTPNMAIVDAELMMKM |
| Template | PKHVTADTGMDVLTHAIEAYVSNMANDYTDGLAMKAIQLV |
| Sequence | PKGLTAYSGIDALVNSIEAYTSVYASEYTNGLALEAIRLI |
| Template | FEYLPRAYQNG-ADELAREKMHNASTIAGMAFANAFLGIN |
| Sequence | FKYLPEAYKNGRTNEKAREKMAHASTMAGMASANAFLGLC |
| Template | HSLAHKLGAEFHIPHGRANTILMPHVIRYNAA-KPKK--- |
| Sequence | HSMAIKLSSEHNIPSGIANALLIEEVIKFNAVDNPVKQAP |
| Template | -----YFKADQRYAEIARMLGL--PAR----TTEEGVESL |
| Sequence | CPQYKYPNTIFRYARIADYIKLGG---NTDE---EKVDLL |
| Template | VQAIIKLA-KQLDMPLSIEACG--VSKQEFESKVEKLAEL |
| Sequence | INKIHELK-KALNIPTSIKDAGVL--EENFYSSLDRISEL |
| Template | AFEDQCTTANPKLPLVSDLVHIYRQAFKGV- |
| Sequence | ALDDQCTGANPRFPLTSEIKEMYINCFKKQP |

^a^ The structure of *Geobacillus thermoglucosidasius* ADH (PDB ID = 3ZDR) was used as the template of homology modeling, and the sequence of its part used in the modeling was denoted. The regions without backbone information was underlined.

^b^ The sequence of *C. acetobutylicum* ADH mapped to the structure of *G. thermoglucosidasius* ADH.
