# Supplementary material for: Engineering Clostridial Aldehyde/Alcohol Dehydrogenase for Selective Butanol Production
Source: mBio. 2019 Jan 22;10(1):e02683-18. doi: 10.1128/mBio.02683-18 (PMC6343042; doi:10.1128/mBio.02683-18)
Supplement: TABLE S2 [file mBio.02683-18-st002.docx]

**Table S2** Comparison of metabolite productions by newly engineered *C. acetobutylicum* strains

|  | Metabolites (g/L) | | | | | Butanol selectivity (g/g)^c^ | B/E ratio (g/g)^d^ |
| --- | --- | --- | --- | --- | --- | --- | --- |
| Strain | Butanol | Acetone | Ethanol | Butyrate | Acetate |  |  |
| M5 (pTHL1-F716L)^a^ | 10.31 _± 0.02_ | 0 | 0.59 _± 0.01_ | 3.38 _± 0.58_ | 7.39 _± 0.48_ | 0.95 | 17.47 _± 0.37_ |
| M5 (pTHL1-N655H)^a^ | 9.71 _± 0.01_ | 0 | 0.61 _± 0.00_ | 3.09 _± 0.07_ | 7.07 _± 0.08_ | 0.94 | 15.91 _± 0.02_ |
| M5 (pTHL1-M572V)^b^ | 0.39 | 0 | 0.16 | 18.94 | 2.43 | 0.71 | 2.43 |
| M5 (pTHL1-S735H)^a^ | 10.50 _± 0.01_ | 0 | 2.42 _± 0.00_ | 0.40 _± 0.01_ | 5.69 _± 0.01_ | 0.81 | 4.33 _± 0.00_ |
| M5 (pTHL1-S712F)^b^ | 0.21 | 0 | 0.91 | 19.53 | 2.96 | 0.18 | 0.23 |
| M5 (pTHL1-I725H)^a^ | 9.86 _± 0.26_ | 0 | 2.05 _± 0.07_ | 0.82 _± 0.03_ | 5.22 _± 0.08_ | 0.82 | 4.81 _± 0.05_ |

^a^ Batch fermentations were conducted in duplicates for reproducibility check.

^b^ As these mutations showed little or non-significant changes in butanol and ethanol production, the fermentations were not conducted in duplicate.

^c^ Butanol selectivity is defined as the ratio of butanol to total solvents (g/g).

^d^ B/E is the ratio of butanol to ethanol (g/g).
