# Supplementary material for: Engineering Clostridial Aldehyde/Alcohol Dehydrogenase for Selective Butanol Production
Source: mBio. 2019 Jan 22;10(1):e02683-18. doi: 10.1128/mBio.02683-18 (PMC6343042; doi:10.1128/mBio.02683-18)
Supplement: TABLE S3 [file mBio.02683-18-st003.docx]

**Table S3** Comparison of fermentation results by newly engineered *C. acetobutylicum* strains^a^

| Strain | Metabolites (g/L) | | | | | Butanol selectivity (g/g)^b^ | B/E ratio  (g/g)^c^ |
| --- | --- | --- | --- | --- | --- | --- | --- |
|  | Butanol | Acetone | Ethanol | Butyrate | Acetate |  |  |
| HKW (pTHL1-Adhe1) | 10.69 _± 0.32_ | 3.20 _± 0.08_ | 9.33 _± 0.37_ | 0.00 _± 0.00_ | 4.70 _± 0.05_ | 0.46 | 1.15 |
| HKW (pTHL1-N613K) | 9.78 _± 0.58_ | 2.55 _± 0.67_ | 7.98 _± 1.35_ | 0.16 _± 0.23_ | 4.74 _± 0.55_ | 0.47 | 1.23 |
| HKW (pTHL1-M619A) | 10.24 _± 0.98_ | 2.64 _± 0.24_ | 6.61 _± 0.55_ | 0.26 _± 0.04_ | 4.79 _± 0.50_ | 0.52 | 1.55 |
| HKW (pTHL1-M619G) | 11.39 _± 0.32_ | 2.93 _± 0.20_ | 5.28 _± 1.29_ | 0.48 _± 0.22_ | 5.11 _± 0.01_ | 0.58 | 2.15 |
| HKW (pTHL1-Y623L) | 10.79 _± 1.03_ | 3.17 _± 0.07_ | 9.53 _± 0.95_ | 0.00 _± 0.00_ | 4.46 _± 0.22_ | 0.46 | 1.14 |
| HKW (pTHL1-F716L) | 11.81 _± 0.21_ | 3.02 _± 0.12_ | 3.51 _± 0.17_ | 0.47 _± 0.07_ | 5.23 _± 0.02_ | 0.64 | 3.40 |
| HKW (pTHL1-M572V) | 0.00 _± 0.00_ | 0.15 _± 0.14_ | 0.00 _± 0.00_ | 11.16 _± 1.94_ | 4.28 _± 0.78_ | 0.00 | 0.00 |
| HKW (pTHL1-N655H) | 9.67 _± 0.49_ | 2.64 _± 0.88_ | 2.86 _± 0.16_ | 0.22 _± 0.31_ | 5.50 _± 0.55_ | 0.64 | 3.34 |
| HKW (pTHL1-S735H) | 10.72 _± 1.04_ | 2.97 _± 0.09_ | 9.14 _± 0.95_ | 0.14 _± 0.20_ | 4.59 _± 0.69_ | 0.47 | 1.18 |
| HKW (pTHL1-S712F) | 0.00 _± 0.00_ | 0.33 _± 0.11_ | 0.00 _± 0.00_ | 13.29 _± 0.20_ | 3.88 _± 0.21_ | 0 | 0.00 |
| HKW (pTHL1-I725H) | 11.47 _± 0.16_ | 2.57 _± 0.10_ | 10.76 _± 0.54_ | 0.26 _± 0.08_ | 4.86 _± 0.20_ | 0.46 | 1.06 |
| M5 (pTHL1-M619GN655H) | 12.00 _± 0.20_ | 0 | 3.02 _± 0.44_ | 2.30 _± 0.46_ | 9.92 _± 0.40_ | 0.80 | 3.97 |
| M5 (pTHL1-M619GF716L) | 1.86 _± 0.99_ | 0 | 1.36 _± 0.06_ | 20.70 _± 2.76_ | 6.70 _± 0.99_ | 0.56 | 1.36 |
| M5 (pTHL1-N655HF716L) | 0.95 _± 0.03_ | 0 | 1.40 _± 0.03_ | 22.67 _± 0.27_ | 6.20 _± 0.19_ | 0.40 | 0.68 |
| M5 (pTHL1-M619GN655HF716L) | 0 | 0 | 1.33 _± 0.02_ | 23.76 _± 0.99_ | 6.57 _± 0.88_ | 0 | 0 |

^a^ Batch fermentations were conducted in duplicates for reproducibility check.

^b^ Butanol selectivity is defined as the ratio of butanol to total solvents (g/g).

^c^ B/E is the ratio of butanol to ethanol (g/g).
